# Design and Analysis of a Pneumatic Spring Testing System for Precision Manufacturing

**DOI:** 10.3390/ma15031121

**Published:** 2022-01-31

**Authors:** Zhibo Sun, Shaofeng Xu, Li Cheng, Na Wang, Yixuan Wang, Minhui Tian, Qingzhen Zhang

**Affiliations:** 1 Engineering Training Center, Beihang University, Beijing 102206, China; sunzb@buaa.edu.cn (Z.S.); lion_na987@buaa.edu.cn (N.W.); magic_wyx@buaa.edu.cn (Y.W.); 2 School of Automation Science and Electrical Engineering, Beihang University, Beijing 100191, China; 3 The First Financial Support Department, Service Assurance Center of PLA General Hospital, Beijing 100853, China; ada193@126.com; 4 Service Support Center of PLA General Hospital, Beijing 100853, China

**Keywords:** pneumatic spring, vibration isolation test, dynamic model, manufacturing

## Abstract

The vibration isolation effect of the pneumatic spring determines the precision of the manufacturing. In this paper, in order to detect the performance of a pneumatic spring, a multi frequency band testing system with different payload is designed and developed. First, the pneumatic spring structure is analyzed, and the stiffness of the pneumatic spring is obtained based on the ideal gas model, Kelvin–Voigt model, and finite element method. Then, to verify the reliability of the system, a dynamic model of the vibration platform is established. Through an analysis of the simulation using the Simulink environment, critical parameters are determined, and the effective conditions of the vibration isolation are obtained. Based on the results from the simulation and experiment, the transmission rate is around 20% under 40 Hz vibration, and 12% under 100 Hz vibration. The pneumatic spring proves to be effective under vibrations beyond 7 H. This achievement will become an important basis for future research concerning precision manufacturing.

## 1. Introduction

Vibration isolation technology is becoming increasingly important for precision manufacturing [1]. Compared with other spring mechanisms, pneumatic spring technology has the advantages of low natural frequency, variable stiffness rate, and high energy storage [2,3,4,5], and it is more suitable for vertical vibration isolation. In recent years, pneumatic vibration isolators (PVIs) have been widely used in the automatic [6,7], precision instruments, and manufacturing fields [8,9]. In precision industries, the pneumatic isolation platform system is usually supported by several PVIs to attenuate vibrations transmitted from the vibration source to the platform.

At present, the PVI system is divided into the passive mode, active mode and active-passive integrated mode. Rubber Passive PVIs are developed with a simple structure and show good performance under high-frequency vibration. The passive isolation method with high-static-low-dynamic stiffness can overcome the dichotomy between low stiffness and load bearing capacity [10]. Typical equivalent mechanical models are established to analyze the vertical behavior of pneumatic springs, such as the Nishimura model [11], Simpack-based model, Vampire-based model [12], and the Berg model [13,14]. Rubber presents nonlinear and hysteresis characteristics in a dynamic response analyzed by Berg. Zhu et al. proposed a dynamic model of the pneumatic spring with an improved friction model, obtaining an accurate prediction of its dynamic characteristic [15]. Chen et.al refined the stiffness model of the Rolling lobe air spring (RLAS) [16]. Quaglia and Sorli [17] presented a dimensionless air spring model. Lee proposed a dynamic model derived from the energy conservation law [18]. M.M. Moheyeldein analyzed the parameters of passive suspensions and investigated the influences of air spring model parameters on vehicle dynamics [19]. N.Y.P. Vo researched the passive quasi-zero stiffness pneumatic spring structure and established the adaptive pneumatic vibration isolation platform [20].

To achieve the requirements of precision manufacturing, a novel pneumatic spring structure is presented in this paper to limit the nonlinear characteristic of rubber. The stiffness of the pneumatic spring is obtained based on the ideal gas model [21], Kelvin–Voigt model [22], and finite element method [23]. Then, to verify the dynamic model, a multi-band frequency and variable load testing platform is established. Compared with the simulation results from Simulink, the testing platform is proved to be reliable. Finally, a summary of the results and conclusions is presented.

## 2. Structure of the Pneumatic Spring

### 2.1. Structure Parameters of the Pneumatic Spring

**Figure 1:**
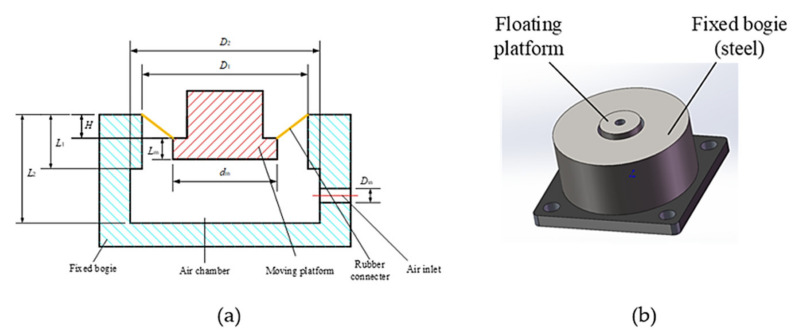
the novel pneumatic spring. (**a**) sketch of the pneumatic spring, (**b**) 3D model of the pneumatic spring.

Compared with the RLAS model, the novel pneumatic spring presents a simple structure. It is comprised of a fixed bogie, a floating platform, an air chamber, an air inlet, and a rubber connector, as can be seen in Figure 1. The fixed bogie is a rigid part connected with the vibration equipment with a flange. The floating platform is also a rigid component to support the load from above. The floating platform is connected to the fixed bogie with the rubber connector, which performs a conical surface. The air chamber is an envelope space surrounded with the fixed bogie, rubber connector and floating platform. The air inlet is a hole in the fixed bogie for air pressure adjustment.

### 2.2. Parameters of the Pneumatic Spring

The vertical stiffness is the key mechanical parameter for dynamic characteristic of the pneumatic spring. The main parameters of the pneumatic spring could be simplified as shown in Figure 1. *L*_1_ is the step length of the air chamber, *L*_2_ denotes the length of the air spring cavity, *D*_1_ and *d_m_* are the outer and inner diameters of the rubber connector, *D*_2_ is the inner diameter of the fixed bogie, and *l_m_* is the length of the floating displacement inside the air chamber. *H* is the floating height. The stiffness model is a parallel combination of two parts: the stiffness model of internal compressed air and the stiffness model of rubber connector.

**Figure 2:**
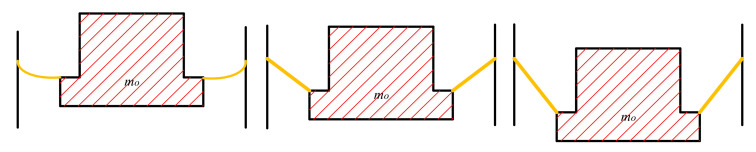
The change of the floating platform.

For the classical model of a pneumatic spring in the literature, the stiffness model of internal compressed air is based on the ideal gas equation [24]:

(1) $$p_{g}V=m_{g}RT$$

where *p_g_* denotes the absolute pressure of the pneumatic spring, *V* is the gas volume in the cavity, *m_g_* is the gas mass, *R* is the molar gas constant, and *T* is the temperature in the cavity. The air condition change determines that it is polytrophic. In a sealed container, the ideal gas equation could be changed to:

(2) $$p_{g}V^{\lambda }=\mathrm{const},$$

where *λ* is the polytrophic coefficient. If the internal temperature is constant, then *λ* = 1. If part of the gas cannot complete the heat exchange process within a short time or there is not much heat exchange and it is regarded as an adiabatic process, then *λ* is recorded as 1.4. As can be seen in the Figure 2, the parameters of the pneumatic spring determine the volume of the air chamber. The volume of the air spring could be divided into two parts: the constant part and the variable part. The constant part could be calculated as:

(3) $$V_{con}=\frac{\pi D_{1}{}^{2}L_{1}+\pi D_{2}{}^{2}L_{2}-\pi d_{m}{}^{2}l_{m}}{4}$$

where *L*_1_, *L_2_*, *D*_1_, *D*_2_, *l_m_* and *d_m_* are the geometric parameters of the pneumatic spring mentioned before. As can be seen in Figure 2, the volume of the air chamber changes with the floating height *H*. The variable part could be approximately calculated as:

(4) $$V_{\mathrm{var}}=\left\{\begin{array}{c} \int_{0}^{H}\pi {\left(\frac{d_{m}}{2}+\left(\frac{D_{1}-d_{m}}{2H}\right)h\right)}^{2}dh\;\;H>0\\ -\int_{H}^{0}\pi {\left(\frac{d_{m}}{2}+\left(\frac{D_{1}-d_{m}}{2H}\right)h\right)}^{2}dh\;\;H\leq 0 \end{array}\right.$$

Through the integral, the variable volume could be simplified as:

(5) $$V_{\mathrm{var}}=\frac{\pi H}{12}\left(D_{1}{}^{2}+D_{1}d_{m}+d_{m}{}^{2}\right)$$

The total volume could be calculated as:

(6) $$V=V_{con}+V_{\mathrm{var}}=\frac{\pi D_{1}{}^{2}L_{1}+\pi D_{2}{}^{2}L_{2}-\pi d_{m}{}^{2}l_{m}}{4}+\frac{\pi H}{12}\left(D_{1}{}^{2}+D_{1}d_{m}+d_{m}{}^{2}\right)$$

To analyze the relationship of the parameters, new index *ε*_1_, *ε*_2_, *ε*_3_, *ε*_4_ are introduced in the model. *ε*_1_ = *D*_1_/*D*_2_, *ε*_2_ = *d_m_*/*D*_1_, *ε*_3_ = *L*_1_/*L*_2_, *ε*_4_ = *l_m_*/*L*_1_. In addition, Equation (6) could be simplified to:

(7) $$V=\frac{\pi D_{1}{}^{2}}{12}\left(3L_{1}\left(1+\frac{1}{\varepsilon_{1}{}^{2}\varepsilon_{3}}-\varepsilon_{2}{}^{2}\varepsilon_{4}\right)+H\left(1+\varepsilon_{2}+\varepsilon_{2}{}^{2}\right)\right)$$

The derivative of the pressure *p_g_* with respect to the height *H* could be derived from Equation (2):

(8) $$\frac{\partial P_{g}}{\partial H}V^{\lambda }+P_{g}\lambda V^{\lambda -1}\frac{\partial V}{\partial H}=0$$

And the derivative is obtained as:

(9) $$\frac{\partial P_{g}}{\partial H}=-\frac{P_{g}\lambda }{V}\frac{\partial V}{\partial H}=-\frac{\pi D_{1}{}^{2}\lambda P_{g}\left(\varepsilon_{2}{}^{2}+\varepsilon_{2}+1\right)}{12V}$$

The force produced by the pneumatic spring could be described as:

(10) $$F_{g}=\left(P_{g}-P_{0}\right)\cdot \frac{\pi }{4}D_{1}{}^{2}$$

where *p*_0_ is the pressure of the atmosphere. In this pneumatic spring model, the effective area is defined as *A_ef_* = *πD*_1_^2^/4. Referring to Equations (9) and (10), the stiffness of the air can be expressed as:

(11) $$k_{g}=\frac{\partial F_{g}}{\partial H}-\frac{P_{g}\lambda A_{ef}{}^{2}\left(\varepsilon_{2}{}^{2}+\varepsilon_{2}+1\right)}{4V}$$

### 2.3. Parameters Model of the Rubber Connector

In this pneumatic spring, the connector between the pneumatic cavity and the fixed bogie is made of rubber. The rubber is an important component of the spring and performs nonlinear characteristic [25]. The stiffness of the rubber connector mainly affects the boundary stiffness.

**Figure 3:**
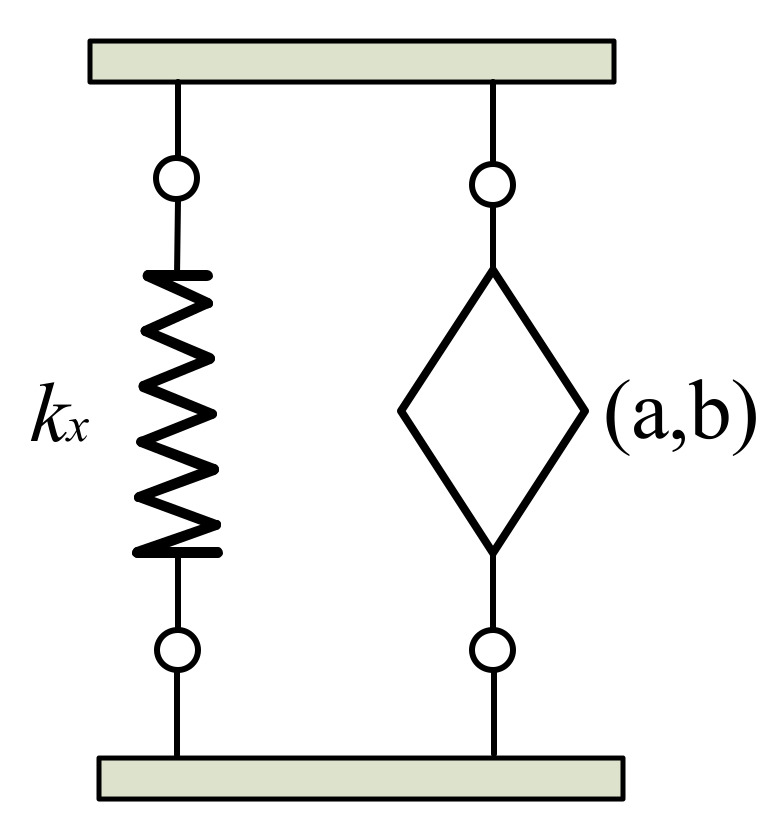
Fractional Kelvin–Voigt model.

Based on the Kelvin–Voigt model, the rubber connector is constituted of elastic stiffness *k_x_* and dash pot in parallel, as shown in Figure 3. 

The expression between force and displacement from the Kelvin–Voigt model from [23] is as follows:

(12) $$k_{r}=k_{x}+bD^{a}$$

where *k_r_* is vertical stiffness of the rubber; *D^a^* is the fractional differential form, *a* is the order of the fractional derivative, *b* is the fractional damping coefficient. By applying the definition of Grünwald fractional derivative [26], the *bD^a^* could be written as follows:

(13) $$D^{a}x\left(t\right)=\underset{\Delta t\to 0}{\lim}\left(\Delta t^{-a}\sum_{i=0}^{N}B_{i+1}f\left(t-i\Delta t\right)\right)$$

where $B_{i+1}={\left(-1\right)}^{i}\left(\begin{array}{c} a\\ i \end{array}\right)=\frac{\Gamma \left(i-a\right)}{\Gamma \left(-a\right)\Gamma \left(i+1\right)}$ and *N* is the interger, *x*(*t*) is the displacement versus time. Additionally dynamic stiffness and phase angle of the fractional Kelvin–Voigt model can be achieved from the Fourier transform of:

(14) $$k_{r}=\sqrt{{\left(k_{x}+b\omega^{a}\cos\left(\frac{a\pi }{2}\right)\right)}^{2}+{\left(b\omega^{a}\sin\left(\frac{a\pi }{2}\right)\right)}^{2}}$$

where *ω* means the amplitude, and loss angle tangent tan*φ* could be calculated as:

(15) $$\tan\varphi =\frac{b\omega^{a}\sin\left(\frac{a\pi }{2}\right)}{k_{x}+b\omega^{a}\cos\left(\frac{a\pi }{2}\right)}$$

**Figure 4:**
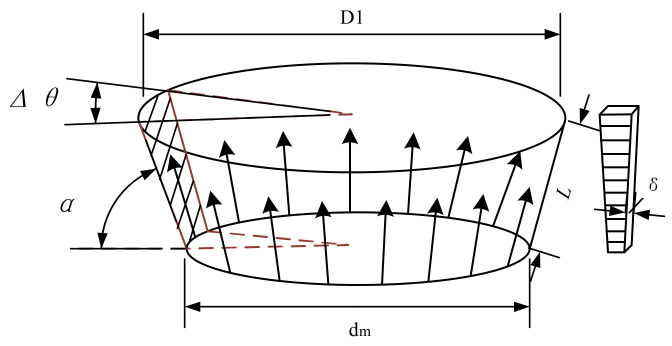
The parameter model of the rubber connector.

The vertical elastic stiffness of the rubber *k_x_* could be analyzed based on the finite element. In the pneumatic spring structure, rubber shapes a conical surface. It is assumed that the rubber material presents a smooth characteristic. Based on the finite element analysis, the rubber connector could be divided into small elements. As shown in Figure 4, the rubber connector is composed of the parallel element rubber stick. Each stick could be recognized as a series of rubber pieces. The elastic stiffness of the rubber connector is a series and parallel structure of the rubber pieces.

It is assumed that the rubber material is smooth. Based on the material mechanics, the stiffness of the rubber can be integrated as:

(16) $$k_{x}=\int_{0}^{2\pi }\frac{\sin\alpha }{\int_{0}^{L}\frac{dl}{E\cdot dS}}=\int_{0}^{2\pi }\frac{\sin\alpha }{\int_{0}^{L}\frac{dl}{E\cdot \left(\frac{D_{1}l-d_{m}l+d_{m}L}{L}\right)\delta \cdot \Delta \theta }}$$

where *E* denotes the Young’s modulus of the rubber, *L* means the elongation length of the rubber and *H* = L*sinα, *δ* is the thickness of the rubber. Through integral, the stiffness of the rubber could be calculated as:

(17) $$k_{x}=-\frac{E\delta \times \pi \times \left(\frac{1}{\varepsilon_{2}}-1\right)d_{m}H}{\left(H^{2}+{\left(\frac{D_{1}-d_{m}}{2}\right)}^{2}\right)\times \log\frac{1}{\varepsilon_{2}}}$$

## 3. Establishment of the Pneumatic Spring Testing System

### 3.1. Structure of the Testing System

**Figure 5:**
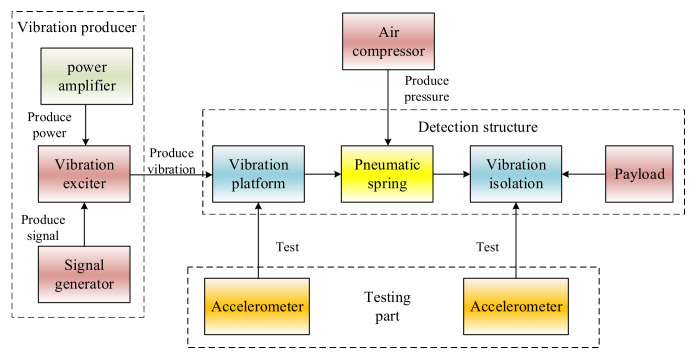
Flow chart of the testing system.

To obtain vibration isolation performance of pneumatic spring, a testing system is developed. In this system, the testing platform with multi-band frequency and payload is established. As can be seen in Figure 5, the platform is mainly composed of four assemblies: vibration producer, testing device, air compressor, and main structure. The vibration producer part includes vibration exciter, power amplifier and signal generator, which provides vibration with frequency from 0 to 20 kHz as the vibration source of the system. The air compressor provides the air pressure for the pneumatic spring. Two accelerometers are applied for vibration tests of the platform as the testing part.

**Figure 6:**
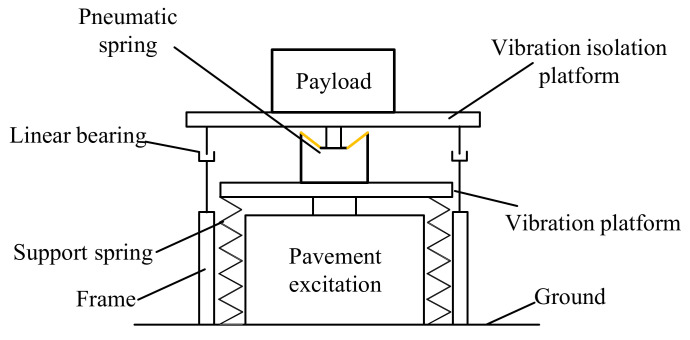
The schematic diagram of detection structure.

The detection structure is the main assemble of the system as shown in Figure 6.

### 3.2. Dynamic Analysis of the Testing System

**Figure 7:**
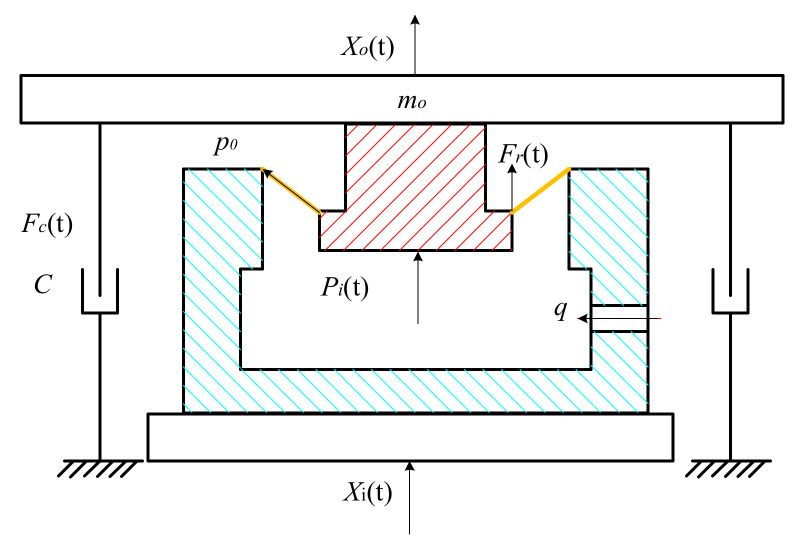
Force analysis of the testing system.

Force analysis of the pneumatic spring model is presented in Figure 7. Pavement vibration acts on the vibration platform directly. The model of the linear bearing is taken into account as damping resistance. The force between the two platforms consists of three parts and could be obtained as:

(18) $$F\left(t\right)=F_{c}\left(t\right)+F_{r}\left(t\right)+F_{p}\left(t\right)$$

where *F*(*t*) means the whole force between the two platforms, *F_c_*(*t*) means the frictional resistance of the linear bearing, *F_r_*(*t*) denotes the nonlinear force from the rubber connector, *F_p_*(*t*) is the pneumatic force produced by the air pressure. *F_c_*(*t*) could be calculated as:

(19) $$F_{c}\left(t\right)=-2f\cdot m_{0}g\frac{{\dot{X}}_{0}\left(t\right)}{\left|{\dot{X}}_{0}\left(t\right)\right|}$$

where *m*_0_ is the mass of payload. *f* is the friction coefficient of the linear bear. *X*_0_(*t*) and *X_i_*(*t*) are the displacement of the vibration isolation platform and the vibration platform. The floating height *H* could also be expressed as:

(20) $$H=L_{2}-\left(X_{i}\left(t\right)-X_{0}\left(t\right)\right)$$

The rubber connector has two states. *h*_0_ is the activating length of the rubber connector. When the floating height *H* reaches the activating length, the elastic stiffness *k_x_* is active. Otherwise the elastic stiffness is not active. The force of the rubber connector could be expressed as:

(21) $$F_{r}\left(t\right)=\left\{\begin{array}{c} -\int_{0}^{H}bD^{a}hdh\;\;H\leq h_{0}\\ -\int_{0}^{H}\left(k_{x}\left(h-h_{0}\right)+bD^{a}h\right)dh\;\;H>h_{0} \end{array}\right.$$

*F_p_*(*t*) could be obtained by the product of the stiffness *k_g_* and the length change of the air chamber *X*_0_(*t*) − *X_i_*(*t*), which could be expressed as:

(22) $$F_{p}\left(t\right)=\int k_{g}\left(X_{0}\left(t\right)-X_{i}\left(t\right)\right)dx$$

where *X*_0_(*t*) and *X_i_*(*t*) are the displacement of the vibration isolation platform and the vibration platform. The floating height *H* could also be expressed as:

(23) $$H=L_{2}-\left(X_{i}\left(t\right)-X_{0}\left(t\right)\right)$$

In the pneumatic system, an air inlet is the key component of air spring damping. According to hydrodynamics and the flow characteristics of the air inlet, the damping of the pneumatic spring can be expressed as:

(24) $$c={\dot{m}}_{g}=sv\sqrt{2g\rho \left({\dot{p}}_{q}-{\dot{p}}_{g}\right)}$$

where *s* is the area of the air inlet, *v* is the air velocity through the air inlet, *g* is the gravitational acceleration, *ρ* is the air density, and *p_q_* is the air pressure out of the air inlet. In the passive spring, the air velocity through the air inlet is zero, so the damping of the spring will be ignored. Based on the Newton–Euler equation, the dynamic model of the system could be established as:

(25) $$F\left(t\right)=m_{0}{\ddot{X}}_{0}\left(t\right)$$

### 3.3. Natural Frequency Analysis of the Testing System

Based on the dynamic analysis, this is the free vibration of coulomb damping system. Coulomb damping has no effect on the natural frequency of the simple harmonic vibration, the natural frequency would be obtained as:

(26) $$\omega =\frac{1}{2\pi }\sqrt{\frac{k}{m_{0}}}$$

where *k* is the stiffness of the system, and could be expressed as:

(27) $$k=k_{g}+k_{r}$$

### 3.4. Vibration Transmission Rate Analysis

The vibration isolation effect of a nonlinear vibration isolation system is evaluated using its transmission rate, which is defined as the ratio of corresponding vibration energy before and after the vibration isolation system [27]. The expression can be presented as:

(28) $$T_{d}=\sqrt{E\left[{\dot{x}}^{2}\right]/E\left[{\dot{x}}_{b}{}^{2}\right]}$$

where $E\left[{\dot{x}}^{2}\right]$ and $E\left[{\dot{x}}_{b}{}^{2}\right]$ denote the mean square of the frame and pavement velocity.

## 4. Design of the Pneumatic Spring Testing System

**Figure 8:**
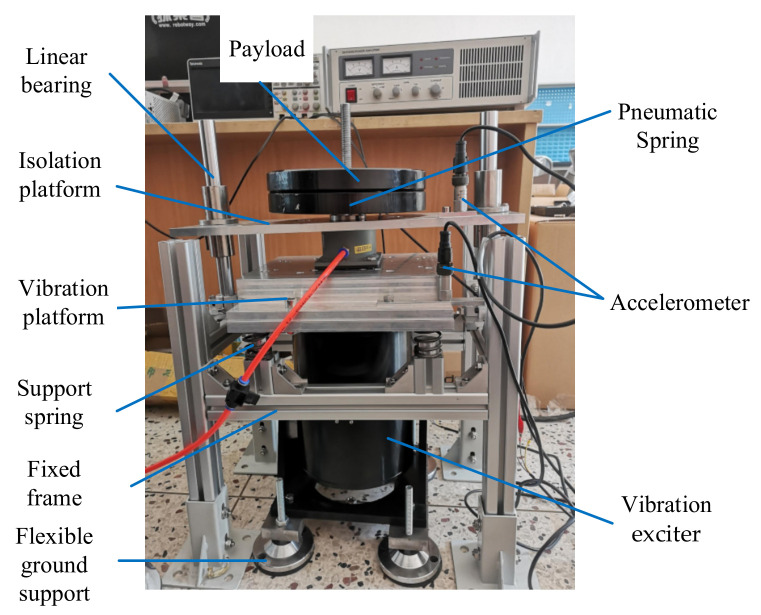
The prototype of the pneumatic spring testing system.

A pneumatic spring testing prototype is designed and developed. As can be seen in Figure 8, the prototype system consists of 10 parts. A vibration exciter provides the vibrations for the system. The main structure consists of payload, vibration isolation platform, linear bearings, pneumatic spring, vibration platform and support springs, and linear bearings. To protect the pavement excitation, four linear springs are applied to share the load from the whole platform. Two linear bearings produce the damping of the system and ensure the transitive vibration direction is vertical. The vibration platform and isolation platform are detected to prove the performance of the pneumatic spring. In addition to the parts above, four flexible ground supports with rubber pads support the vibration exciter to limit the vibration transmitted to the ground.

The testing system could realize the parameter detection of a single or two parallel pneumatic springs under multi-band frequency and different load conditions:

(1) The vibration platform and the isolation platform are designed with a single or two parallel connectors for three kinds of pneumatic springs. The system could test the performance of different combinations of the springs.
(2) Each load on the isolation platform equals 10 kg, and the payload of the system could change from 0 to 50 kg. The system could test the performance of the springs under different payloads.
(3) The air pressure of the spring and the frequency of the simulated vibration are variable. The system could test the performance of the springs under different conditions.

## 5. Comparison between Simulation and Experiment of the Testing System

### 5.1. Results of the Simulation

Based on the dynamic model, the simulation of the testing system is established under Matlab/Simulink environment. The initial parameters of the pneumatic spring are presented in Table 1.

Five computing modules are established, which are dynamic analysis, natural frequency analysis, transmission rate analysis, rubber stiffness analysis, and friction resistance analysis. In this simulation, three parameters include *ε*_2_, *p*_g_ and *m*_0_ are set at input variables. The evaluating values such as vibration velocity, natural frequency and transmission rate are the outputs of the system.

**Figure 9:**
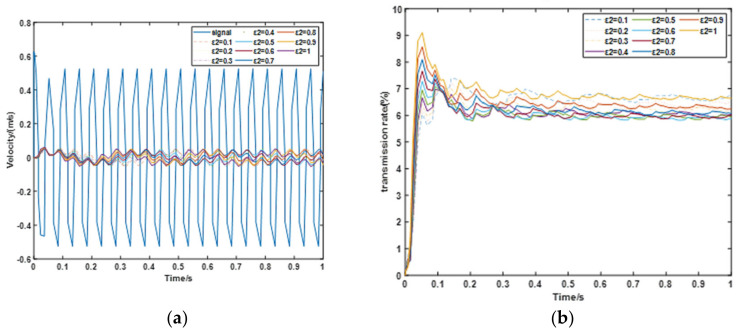
Velocity and transmission analysis of the first simulation. (**a**) Comparison of the velocity and (**b**) Comparison of the transmission rate.

The first simulation analyzes the *ε*_2_ effect of the system. In this simulation, the payload is set at 30 kg. The frequency and the amplitude of the vibration from the exciter are 20 Hz and 5 mm respectively, and the simulation time is 1 s. From [28], the air pressure is set at 1.6 bar, As can be seen in the comparison with Figure 9, the maximum amplitude of the velocity from excitation source is 1.154 m/s, and vibration is greatly reduced through the PVI. The transmission rate is below 10% in the process.

**Figure 10:**
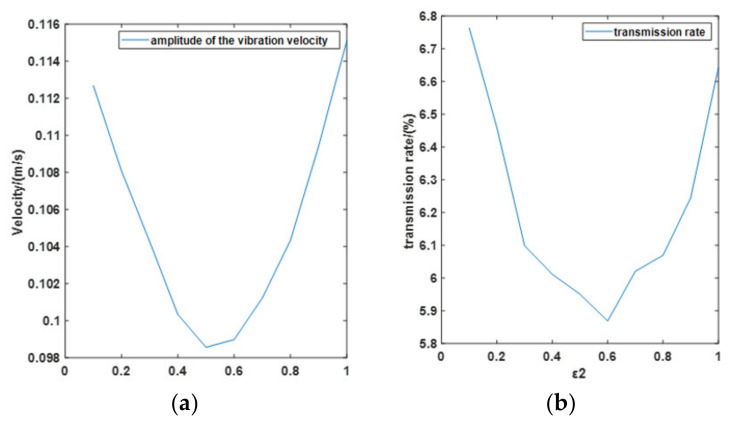
*ε*_2_ effect under the simulation. (**a**) Velocity change with *ε*_2_ and (**b**) Transmission rate change with *ε*_2_.

As can be seen in Figure 10, compared with other results, *ε*_2_ = 0.5 shows the best performance in the velocity reduction, and *ε*_2_ = 0.6 shows best performance in the transmission rate.

**Figure 11:**
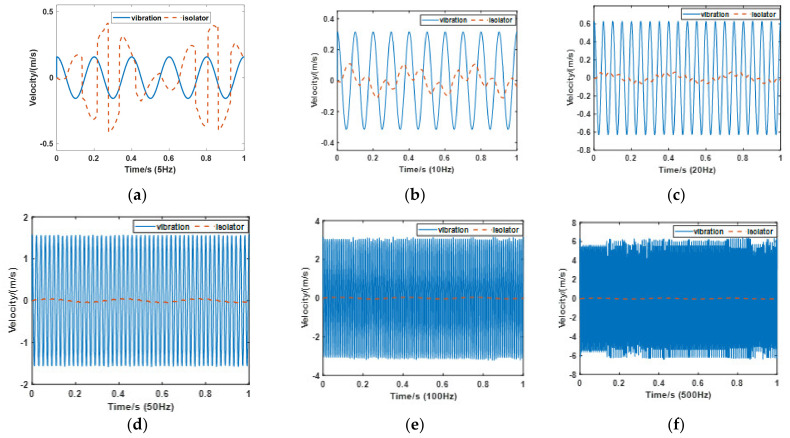
Isolator performance under different frequency vibration in the second simulation. (**a**) 5 Hz; (**b**) 10 Hz; (**c**) 20 Hz; (**d**) 50 Hz; (**e**) 100 Hz and (**f**) 500 Hz.

**Figure 12:**
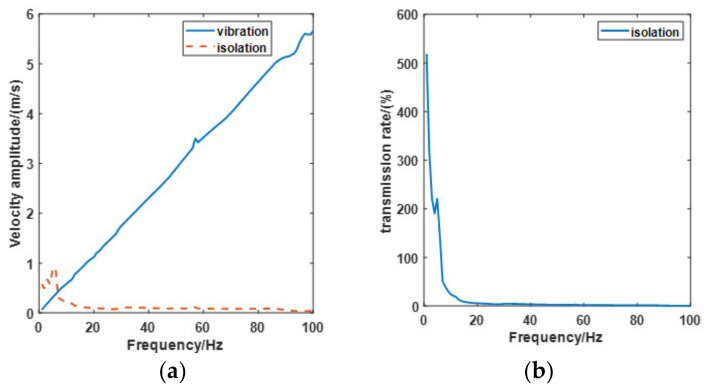
frequency effect of the simulation. (**a**) Velocity amplitude change and (**b**) Transmission rate change.

The second simulation analyzes the vibration frequency effect of the system. In this simulation, the payload and air pressure are also set at 30 kg and 1.6 bar. The amplitude of the vibration is 5 mm, and the simulation time is 1 s. Based on the first simulation, *ε*_2_ is determined to be 0.5. Frequencies of the vibration are set at 5 Hz, 10 Hz, 20 Hz, 50 Hz, 100 Hz, and 500 Hz. Figure 11 shows the performance of the PVI under each vibration. Compared with other results, on the condition of 5 Hz vibration, the pneumatic spring no longer has any effect on vibration isolation. For further analysis, 1 Hz to 100 Hz vibrations are analyzed with 1 Hz steps, as can be seen in Figure 12. From the transmission rate results, we can see that the pneumatic spring performs effectively (with a transmission rate below 100%) only when the vibration frequency raises above 6 Hz. The velocity amplitude tends to be stable when the vibration frequency is beyond 20 Hz, and the stable value is around 0.1 m/s. The transmission rate drops to 5.9% under the 20 Hz vibration.

Based on the results of the simulations before and the experimental setup condition, the third simulation analyzes the performance of the relationship between the payload, vibration frequency, and the absolute pressure. In this simulation, *ε*_2_ is set at 0.5. Vibration frequency ranges from 20 to 100 Hz, and the step size is 20. The payload ranges from 10 to 50 kg with step size 10. The air pressure ranges from 1.2 to 2 bar with step size 0.2.

**Figure 13:**
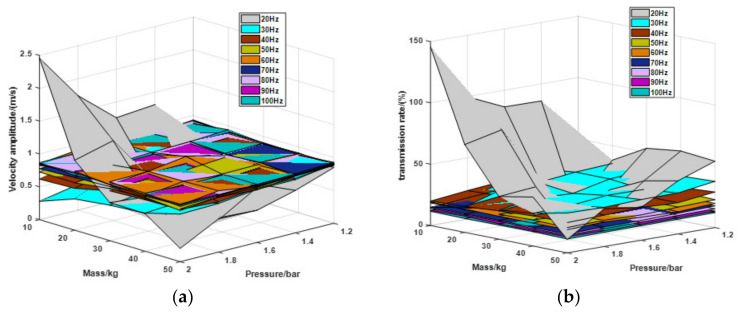
Results under different conditions in the third simulation. (**a**) Velocity amplitude change and (**b**) Transmission rate change.

As can be seen in the comparison in Figure 13, velocity amplitudes remain largely the same under the 40 to 100 Hz vibration. Under 20 Hz vibration, the different pressures and loads has a great impact on the vibration isolation performance. The velocity amplitude drops to 0.117 m/s with payload 20 kg and air pressure 1.4 bar and raises to 2.458 m/s with payload 10 kg and air pressure 2 bar. As the vibration frequency increases, the transmission rates of the vibration decrease. When the frequency raises to 100 Hz, the transmission rate drops to about 11.5%.

From the simulations above, the following conclusions can be drawn: (1) the key parameter *ε*_2_ is optimized as 0.5 for low velocity amplitude. (2) The pneumatic spring performs effectively only when the vibration frequency raises above 6 Hz. (3) Under 10 to 50 kg payload and 1.2 to 2 bar air pressure, the vibration isolation performs stable effect with 40 to 100 Hz vibration, and the transmission rate drops down with the vibration frequency increases.

### 5.2. Results of the Experiment

Corresponding to the simulation condition above, the payload mass of the isolation platform is set at 10 and 50 kg. The pressures of the experiment are set at 1.2 and 2 bar. The vibration frequency is set at 40 and 100 Hz, respectively.

**Figure 14:**
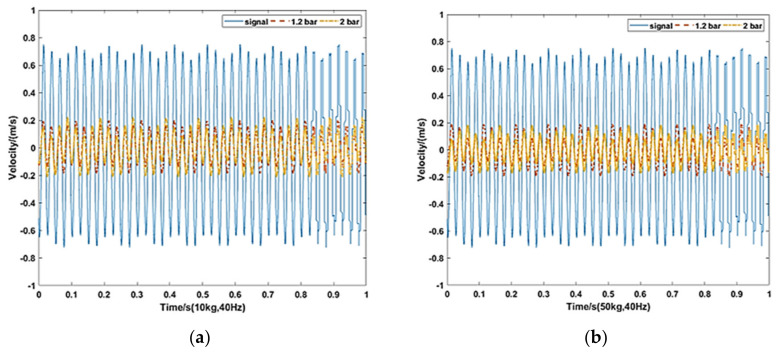
Experimental results under 40 Hz vibration. (**a**) 10 kg, 40 Hz and (**b**) 50 kg, 40 Hz.

As can be seen in Figure 14, the results show that the pneumatic spring performs well under the 40 Hz vibration with 10 and 50 kg payload. The maximum velocity amplitude is 1.462 m/s from the vibration source. Through the pneumatic spring the maximum velocity amplitudes are 0.3952, 0.4373, 0.3842 and 0.3713 under 10 and 50 kg with 1.2 and 2 bar absolute pressure respectively. The transmission rates under 100 Hz vibration are 23.6%, 24%, 21.4%, and 20.1%, respectively.

**Figure 15:**
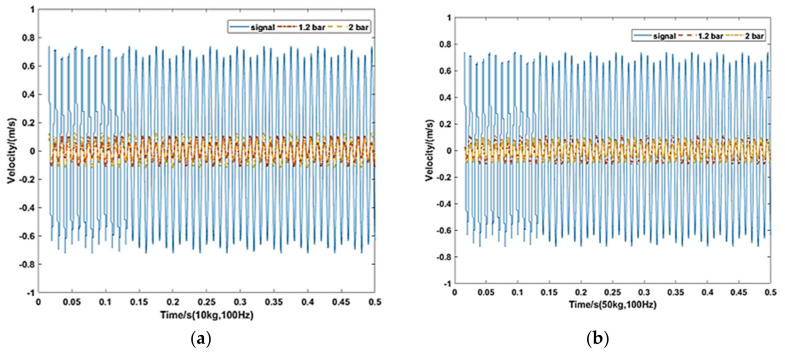
Experimental results under 100 Hz vibration. (**a**) 10 kg, 100 Hz and (**b**) 50 kg, 100 Hz.

As can be seen in Figure 15, Under the 100 Hz vibration, the maximum velocity amplitude is 1.475 m/s from the vibration source. Through the pneumatic spring the maximum velocity amplitudes are 0.2210, 0.2565, 0.2168, and 0.1988 under 10 and 50 kg with 1.2 and 2 bar absolute pressure, respectively. The transmission rates under 40 Hz vibration are 12.6%, 14.3%, 12.8%, and 11.3%, respectively.

To prove the effective application range of pneumatic spring, another experiment is made. In this experiment, the transmission rate of spring is tested under 3 Hz, 7 Hz and 10 Hz vibration with 10 kg load and 1.2 bar pressure. The results of the simulation and the experiment are shown in Table 2.

Compared with the simulation, the results of the experiment prove that:

(1) All the error rates of the simulation and experiment are below 20%. The pneumatic spring testing system is proved to be reliable the dynamic model and the simulation model are effective and could be applied to analysis of the pneumatic spring with the same structure.
(2) The transmission rate is around 20% under 40 Hz vibration, and 12% under 100 Hz vibration. The pneumatic spring is suitable when the vibration environment above 40 Hz, and performs better with vibration frequencies under 100 Hz. That proves the higher-frequency vibration produced, the better performance the spring shows.
(3) From the transmission rate results under 10 Hz, 7 Hz, and 3 Hz, the pneumatic spring proves to be effective under the vibration beyond 7 H. For low-frequency vibrations such as 3 Hz, this pneumatic spring loses effectiveness.

## 6. Conclusions

(1) A pneumatic spring with conical rubber connector is presented for precision manufacturing. The stiffness of the pneumatic spring is analyzed based on the ideal gas model, Kelvin–Voigt model and finite element method. The dynamic model of the pneumatic spring is established as the basic of the simulation.
(2) A multi frequency band testing system with different payload is designed and established to detect the performance of the pneumatic spring. Based on the structure of the platform, the performance of the system is simulated with the Matlab/Simulink environment. Through the simulation, the critical parameter *ε*_2_ is determined to be 0.5, the suitable vibration frequency is beyond 40 Hz.
(3) A comparison between simulation and the experiment is obtained. All the error rates between the simulation and experiment are below 20%. The results prove that: the transmission rate is around 20% under 40 Hz vibration, and 12% under 100 Hz vibration. The pneumatic spring performs well in vibration isolation. The pneumatic spring proves to be effective under the vibration beyond 7 H based on the simulation and experiment results.

RLAS performs good in vibration isolation when the frequency is beyond 10 Hz. Compared with the RLAS model in [20], the novel PVI with a simple structure could limit the nonlinear influence of rubber and shows better performance under 7–10 Hz vibration. Compared with other setups in [17], the structure of the testing system is simple and presents wider functions. It could test the performance of different combinations of the springs under different loads with different frequency. The equipment demonstrates better functionality for pneumatic spring testing. For future research, this structure can be converted into an active suspension for low-frequency vibration isolation. If so, this would become an important basis for improving precision manufacturing with low frequency vibrations and a fundamental theory for the precise control of pneumatic springs.

## Figures and Tables

**Table 1:** Initial parameters of the simulation.

Parameters	Description (Unit)	Values
*L*_1_	step length of the air chamber (mm)	23
*L*_2_	length of the air spring cavity (mm)	56
*D*_1_	outer diameter of the rubber connector (mm)	80
*D*_2_	inner diameter of the fixed bogie (mm)	86

**Table 2:** Comparison between simulation and experiment.

Condition		Simulation Transmission Rate	Experiment Transmission Rate
40 Hz	10 kg, 1.2 bar	22.1%	23.6%
	10 kg, 2 bar	22.7%	24%
	50 kg, 1.2 bar	20.6%	21.4%
	50 kg, 2 bar	19.3%	20.1%
100 Hz	10 kg, 1.2 bar	11.8%	12.6%
	10 kg, 2 bar	12.1%	14.3%
	50 kg, 1.2 bar	11.5%	12.8%
	50 kg, 2 bar	11.3%	11.3%
10 Hz	10 kg, 1.2 bar	30.0%	35.8%
7 Hz	10 kg, 1.2 bar	90.19%	94.4%
3 Hz	10 kg, 1.2 bar	233.3%	276.8%

## Data Availability

Data is contained within the article.
